# Birth, stillbirth and death registration data completeness, quality and utility in population-based surveys: EN-INDEPTH study

**DOI:** 10.1186/s12963-020-00231-2

**Published:** 2021-02-08

**Authors:** Simon Kasasa, Davis Natukwatsa, Edward Galiwango, Tryphena Nareeba, Collins Gyezaho, Ane Baerent Fisker, Mezgebu Yitayal Mengistu, Francis Dzabeng, M. Moinuddin Haider, Judith Yargawa, Joseph Akuze, Angela Baschieri, Claudia Cappa, Debra Jackson, Joy E. Lawn, Hannah Blencowe, Dan Kajungu, the Every Newborn-INDEPTH Study Collaborative Group

**Affiliations:** 1 IgangaMayuge Health and Demographic Surveillance System, Makerere University Centre for Health and Population Research, Iganga, Uganda; 2 Department of Epidemiology and Biostatistics, Makerere University School of Public Health, Kampala, Uganda; 3 Makerere University Centre for Health and Population Research, Makerere, Uganda; 4 Bandim Health Project, Bissau, Guinea-Bissau; 5 Research Centre for Vitamins and Vaccines, Statens Serum Institut, Copenhagen, Denmark; 6 Departmet of Clinical Research, Open Patient data Explorative Network (OPEN), University of Southern Denmark, Odense, Denmark; 7 Dabat Research Centre Health and Demographic Surveillance System, Dabat, Ethiopia; 8 Department of Health Systems and Policy, University of Gondar, Gondar, Ethiopia; 9 Kintampo Health Research Centre, Kintampo, Ghana; 10 Health Systems and Population Studies Division, icddr,b, Dhaka, Bangladesh; 11 Maternal, Adolescent, Reproductive & Child Health (MARCH) Centre, London School of Hygiene & Tropical Medicine, London, UK; 12 Departent of Health Policy, Planning and Management, Makerere University School of Public Health, Kampala, Uganda; 13 Centre of Excellence for Maternal Newborn and Child Health Research, Makerere University, Kampala, Uganda; 14 United Nations Children’s Fund (UNICEF), New York, USA; 15 School of Public Health, University of the Western Cape, Cape Town, South Africa

**Keywords:** Neonatal death, Stillbirth, Survey, Birth certificates, Birth registration, Death registration, Vital statistics

## Abstract

**Background:**

Birth registration is a child’s first right. Registration of live births, stillbirths and deaths is foundational for national planning. Completeness of birth registration for live births in low- and middle-income countries is measured through population-based surveys which do not currently include completeness of stillbirth or death registration.

**Methods:**

The EN-INDEPTH population-based survey of women of reproductive age was undertaken in five Health and Demographic Surveillance System sites in Bangladesh, Ethiopia, Ghana, Guinea-Bissau and Uganda (2017–2018). In four African sites, we included new/modified questions regarding registration for 1177 stillbirths and 11,881 livebirths (1333 neonatal deaths and 10,548 surviving the neonatal period). Questions were evaluated for completeness of responses, data quality, time to administer and estimates of registration completeness using descriptive statistics. Timing of birth registration, factors associated with non-registration and reported barriers were assessed using descriptive statistics and logistic regression.

**Results:**

Almost all women, irrespective of their baby’s survival, responded to registration questions, taking an average of < 1 min. Reported completeness of birth registration was 30.7% (6.1-53.5%) for babies surviving the neonatal period, compared to 1.7% for neonatal deaths (0.4–5.7%). Women were able to report age at birth registration for 93.6% of babies. Non-registration of babies surviving the neonatal period was significantly higher for home-born children (aOR 1.43 (95% CI 1.27–1.60)) and in Dabat (Ethiopia) (aOR 4.11 (95% CI 3.37–5.01)). Other socio-demographic factors associated with non-registration included younger age of mother, more prior births, little or no education, and lower socio-economic status. Neonatal death registration questions were feasible (100% women responded; only 1% did not know), revealing extremely low completeness with only 1.2% of neonatal deaths reported as registered. Despite > 70% of stillbirths occurring in facilities, only 2.5% were reported as registered.

**Conclusions:**

Questions on birth, stillbirth and death registration were feasible in a household survey. Completeness of birth registration is low in all four sites, but stillbirth and neonatal death registration was very low. Closing the registration gap amongst facility births could increase registration of both livebirths and facility deaths, including stillbirths, but will require co-ordination between civil registration systems and the often over-stretched health sector. Investment and innovation is required to capture birth and especially deaths in both facility and community systems.

## Key findings

**Table:** 

**What is new**
• **What was known already:** Birth registration is a marker of civil rights and is receiving increased investment. Household surveys, including Demographic and Health Surveys (DHS) and UNICEF’s Multiple Indicator Cluster Surveys (MICS), are important sources of population-level information on completeness of birth registration but the data quality is unknown. Stillbirth registration or neonatal/child death registration are not included in DHS or MICS surveys. • **What was done:** As part of the EN-INDEPTH survey, we evaluated new and modified questions on birth, stillbirth and death registration for 13,058 births (1177 stillbirths, 1333 neonatal deaths, 10,548 live births surviving the neonatal period) in four African Health and Demographic Surveillance Systems sites.
**What was found?**
• **Completeness of responses:** Questions were almost universally answered (> 99% responses complete, < 5% do not know responses) in an average of < 1 min in all sites. • **Completeness of registration:** *Birth registration* completeness was 30.7% overall for children surviving the neonatal period (with variation across the four study sites, being lowest in Dabat, Ethiopia), compared to just 1.7% for babies who died in the first 28 days. Most infants were reported to be registered in the first 3 months of life. Completeness of *neonatal death and stillbirth registration* was very low with only 1.2% of babies who died in the neonatal period and 2.5% of stillbirths reported as registered. • **Data quality:** Women reported age at birth registration for 93.6% of registered children surviving the neonatal period, with a plausible distribution of age at registration, but some heaping at 6-month intervals. • **Data utility:** Inequities in birth registrations are clear in this study population with children more likely to be unregistered if they were born at home, had younger or less educated mothers and lower socio-economic status. Common reasons for non-registration amongst 7312 unregistered children surviving the neonatal period were complexity of registration process (36%), financial barriers (28%) and distance (16%).
**What next in measurement and research?**
• **Measurement improvement now:** Reliable measures in surveys are crucial to track birth registration completeness and identify who is left behind in this marker for child rights, e.g., by sex, maternal education, or socio-economic status. Given that around 80% of the world’s births are now in facilities, facilitating facility-based registration for these babies would increase birth and stillbirth registration completeness and also allow tracking through routine facility and vital statistics data, instead of relying only on 5-yearly surveys. • **Research needed:** Death registration for stillbirths and neonatal deaths are extremely low. Further research is needed to identify solutions to address barriers to death registration in facility and community systems.

## Background

Despite the right to an identity being enshrined in the UN convention on the rights of the child [1] as well as in other major human rights instruments, globally millions are born and die each year without ever being officially recorded in a national civil registration system [2, 3]. Failure to be registered is associated with poverty, vulnerability to rights violations, marginalisation and exclusion from health, social, economic and political development [3]. Accurate information on live births, stillbirths and deaths is required for public health tracking improvements in maternal-child health and progress towards Sustainable Development Goals (SDG) 3, 11 and 16 [4, 5]. In theory, civil registration and vital statistics (CRVS) systems are the preferred mechanism for measuring all births (both live and stillbirths) and deaths; however, unfortunately CRVS in the countries with the highest mortality burden have the lowest completeness of birth, stillbirth and death registration [6].

Investment in CRVS systems in many low- and middle-income countries (LMICs) is increasing [6]. Particular attention has been placed on birth registration, which is the term used to refer to registration of live births, leading to substantial improvements over the past decade, with three out of four children under the age of five worldwide now registered with civil authorities [3, 6–9]. The majority (87%) of the estimated 166 million unregistered children under-5 years are in southern Asia and sub-Saharan Africa and wide socio-economic inequalities and gaps between urban and rural areas remain [3, 10].

Death registration systems lag behind birth registration [11]. Only 60 countries worldwide are currently assessed as having good quality overall child death registration data from vital statistics, with few outside developed regions [12]. The status for information on neonatal deaths is even worse, with fewer than 5% of all neonatal deaths worldwide estimated to receive a death certificate [12]. Information on stillbirths is collected within a ‘stillbirth or fetal death register’ within the CRVS system, and whilst not currently systematically collated at a global level, is likely to be worse than for neonatal deaths [13].

Estimates of the completeness of registration data in LMICs rely on nationally representative household surveys such as demographic and health surveys (DHS) and multiple indicator cluster surveys (MICS) [14]. In the registration of vital events, first the event is registered with the civil authorities, and then a certificate is issued. In many settings certificates are not issued for stillbirths, and even for other events, financial and other logistically barriers result in not every registered event receiving a certificate. In DHS, there is a single birth registration question in the household roster asking if children 0–4 years who are currently alive have a birth certificate, with a probe question regarding birth registration with the civil authorities asked only for children without a birth certificate. In MICS similar questions are asked regarding surviving children under five in the household. Whilst completeness of birth certification is critical to protecting the rights of surviving children, not including children who died prior to the survey visit might have overestimated the completeness of birth registration. Neither platform includes questions on birth or death registration for children who have died or questions on registration for stillbirths. Failure to include such questions is potentially a missed opportunity within vital statistics to track completeness of registration for stillbirths, neonatal and child deaths. However, the feasibility of collecting information on birth or death registration for children who have died, or for stillbirths is not known.

This paper is part of a series of papers from the Every Newborn-International Network for the Demographic Evaluation of Populations and their Health (EN-INDEPTH) study in five health and demographic surveillance system (HDSS) sites in Africa and Asia. This paper aims to improve understanding of the measurement of birth, stillbirth and death registration in population-based household surveys through the following objectives:

1. ***Survey question performance:*** Evaluate new and modified questions on birth, stillbirth and neonatal death registration addressed to women in a population-based survey, including completeness of responses and time implications.
2. ***Data utility:*** Assess the information obtained through the survey regarding birth, stillbirth and neonatal death registration including estimates of completeness and timing of registration, factors associated with non-registration and reported barriers to registration.

## Methods

### EN-INDEPTH study design and setting

The EN-INDEPTH study was a cross-sectional multi-site study conducted between July 2017 and August 2018, including a survey of 69,176 women aged 15–49 years undertaken in five HDSS sites: Bandim in Guinea-Bissau, Dabat in Ethiopia, IgangaMayuge in Uganda, Matlab in Bangladesh and Kintampo in Ghana (Additional file 1 provides background details of these sites). The protocol and main study paper are published elsewhere and provide further details [11, 15]. The primary objective of the study was to randomly compare two methods of retrospective recording of pregnancy outcomes in surveys: full birth history with additional questions on pregnancy losses (FBH+) and full pregnancy history (FPH) as detailed elsewhere [11, 15].

Both woman and interviewer data were collected on Android tablets using the Survey Solutions data collection and management system [16]. Interviewers were recruited locally and were familiar with the culture and dialect of the study area. Following completion of data collection, data from the five HDSS sites were anonymised by local HDSS scientists, encrypted and then shared [11].

The EN-INDEPTH study also investigated the performance of existing, modified and new survey questions to capture additional information on pregnancies and birth. This included a sub-sample of survey respondents in the four African sites being asked to provide answers to questions on birth, and where relevant death, registration for their most recent live birth and all neonatal deaths and stillbirths since 1st January 2012 (Additional file 2). Whilst possession of a birth certificate is critical for ensuring the rights of surviving children, in these analyses we are especially interested in whether babies were registered and thus counted in vital statistics to inform public health action. These four countries differ in terms of legal frameworks and operational systems for CRVS (Table 1).

**Table 1:** Comparison of legal framework and operational systems for birth and stillbirth registration

	Ghana	Guinea-Bissau	Ethiopia	Uganda
**Legislation status**	Registration of Births, Deaths Act 1965. Not linked to services	1967 Civil code of registry. Birth certificate required for school enrolment, ID, Passport	Proclamations 2012 and 2017. First permanent, compulsory and universal nationwide system launched in August 2016	2015 Act amended. Certificate issued in 2 days and required for school enrolment, national ID, bank account, passport, driving licence, public service job, joining police and military forces
**Responsibility to notify birth**	Parents, guardian, occupier of premises where birth took place	Parents, doctor, family member, village chief	Parents, doctor, family member	Hospital, sub-county chief, parents/guardian
**Documents required for birth registration**	Biological parents’ ID	IDs of parents, presence of parents, birth notification from hospital, immunization card to verify DOB, Child name	Parents IDs, parents physical presence (unless for justified reason), Child name, DOB	Biological parents’ ID, parents birth certificates, child names, place of birth notification
**Charge for birth registration**	Nil	Free for 0–7-year old. No fee for abridged certificate	Nil	Nil
**Charge for birth certificate**	Nil	Fee detailed birth certificate	Charged	5000 shillings^a^
**Legal required time-frame for registration**	1 year from birth	30 days	90 days	No limit
**Late registration penalty**	Yes, after 1 year. Fine not shown	Yes, 8–13 years and higher for 14 years and more	Fine 5000 Ethiopian Birr^b^ or imprisonment up to 6 months	None for nationals
**Methods for birth registration**	Electronic	Manual	-----	Manual + electronic(computer/tabs)
**Enforcement of law**	-----	-----	-----	Implemented, but challenged with late reporting, corruption, lack of national internet coverage data transition
**Current initiatives to increase birth registration**	From 2010 to 2014 HDSS staff notified births to district levels and encouraged mothers to register births during 1st month of life. Funding for this initiative ended in 2014.	UNICEF sponsors radio spot messages encouraging birth registration; however, there are no direct incentives to register.	Mass media and health worker campaigns to increase community awareness. No direct incentives to encourage birth registration	Some recent innovation using app-based Mobile Vital records System to register births in community and some facilities.
**Stillbirth registration**	Registration of Births and Deaths Act 1965 requires registration of all fetal deaths. There is a separate register for stillborn children.	Fetal deaths (stillbirths) are not registered.	Fetal deaths (stillbirths) are not registered	Fetal deaths (stillbirths) are not registered

Data source: UNICEF [38]; *DOB* date of birth

^a^Around 1.4 US dollars

^b^Around 150 US dollars

### Methods by objective

#### Objective 1: Evaluate survey question performance

Information on birth, stillbirth and death registration was assessed in the survey using the questions shown in Table 2. These questions included standard MICS questions and additional questions on time from birth to registration and for babies surviving the neonatal period reasons for non-registration. Each question was assessed for completeness and ‘don’t know’ responses including any variations by maternal and child characteristics including survival status using descriptive statistics. Numerical answers were assessed for heaping using graphical plots and heaping index.

**Table 2:** Birth and death registration questions in Demographic and Health, and Multiple Indicator Cluster and EN-INDEPTH surveys

	Placement in questionnaire	Target	Coding of responses
**DHS-7 and DHS-8 standard birth and death registration questions**			
Does (name) have a birth certificate? If no, probe: Has (name)'s birth ever been registered with the civil authority?	Household roster	0-4 year olds currently alive	1 = has certificate, 2 = registered, 3 = neither, 4 = don’t know
Question(s) on death registration	None	-	-
**MICS6 birth and death registration questions**			
Does (name) have a birth certificate? If yes, ask: May I see it?	Questionnaire for children under five administered to mothers/ caretaker	0-4 year olds currently alive	1 = yes, seen; 2 = yes, not seen; 3 = no; 4 = don’t know
If no or don’t know to above question: Has (name)’s birth been registered with the civil authorities?^a^	Questionnaire for children under five administered to mothers/ caretaker	0-4 year olds currently alive	1 = yes, 2 = no, 3 = don’t know
If no or don’t know to above question: ‘Do you know how to register (name)’s birth?’	Questionnaire for children under five administered to mothers/ caretaker	0-4 year olds currently alive	1 = yes, 2 = no
Question(s) on death registration	None	-	-
**EN-INDEPTH survey birth and death registration questions**			
Does (name/this baby) have a birth certificate?^a^ If yes, ask: May I see it?	Section 4 of the women’s questionnaire	Subset of livebirths and all stillbirths since 1st January 2012	1 = yes, seen; 2 = yes, not seen; 3 = no; 4 = don’t know
Has (name/this baby)'s birth been registered with the civil authorities?^a^	Section 4 of the women’s questionnaire	Livebirths and stillbirths in last 5 years whose mother’s answered ‘No’ or ‘Don't know’ to previous question	1 = yes, 2 = no, 3 = don’t know
At which age was (name/this baby) registered?	Section 4 of the women’s questionnaire	Livebirths and stillbirths in last 5 years	1 = weeks if less than 4 weeks, 2 = months if less than 2 years, 3 = years if 2 years or more, 4 = don’t know
Specify number of weeks, months or years as appropriate	Section 4 of the women’s questionnaire	Livebirths and stillbirths in last 5 years	Integer
I am interested in knowing about the main reasons why you have not registered (name). I will now read some possible reasons. Please let me know if any apply: (i) Birth registration is not a legal requirement. (ii) There is not enough money to pay the cost of registering (name). (iii) The distance to the registration centre is far. (iv) The registration process is too complicated to understand. (v) I am unable to produce the full set of documents required to register (name). (vi) The father of (name) is required to be present but he is unable or unwilling to attend. (vii) The name of the child is required but it has not yet been given by the family. (viii) Are there other reasons why you did not register (name) that have not already been mentioned?	Section 4 of the women’s questionnaire	Livebirths in last 5 years surviving the neonatal period	For each category: 1 = yes, 2 = no; plus free text box to specify for ‘other reasons’
Does (name/this baby) have a death certificate? If yes, ask: ‘May I see it?’	Section 4 of the women’s questionnaire	Neonatal deaths and stillbirths in last 5 years only	1 = yes, seen; 2 = yes, not seen; 3 = no; 4 = don’t know

^a^These questions use the standard questions and responses for UNICEF’s MICs surveys, except if the baby was stillborn or died before being named the baby was referred to as ‘this baby’. A positive response to either of these questions was taken to indicate that birth registration had occurred.

Time taken to complete the birth and death registration section was assessed using the EN-INDEPTH survey paradata. Paradata were collected by the app during the survey and provide detailed records of data entry and corrections made for each question, stored as time-stamped ‘events’. Time taken for each question was defined as the time interval between the time-stamp for the question(s) under study and the previous question. Missing data and responses that took 30 min or more were excluded.

#### Objective 2: Assess utility of survey data

Completeness of birth registration and mean time from birth to registration were calculated overall and by HDSS site for babies who died before the 28th day after birth (neonatal deaths) and those surviving the neonatal period separately. As specific questions regarding stillbirth registration were not asked, babies who were stillborn (stillbirths) were classified as registered if the mother reported that the baby was registered through either the birth or the death registration questions.

Reported reasons for non-registration of children surviving the neonatal period are presented graphically using descriptive statistics. Factors associated with non-registration of these children were explored using logistic regression, with registration status as the dependent variable. A gap analysis comparing the coverage of facility births to completeness of birth, stillbirth and neonatal death registration was undertaken to examine potential missed opportunities for registration for facility births.

All data management and quantitative analyses were undertaken using Stata 15.1. Results are reported in accordance with STROBE Statement checklists for cross-sectional studies [17] (Additional file 3).

## Results

### Overall

**Fig. 1:**
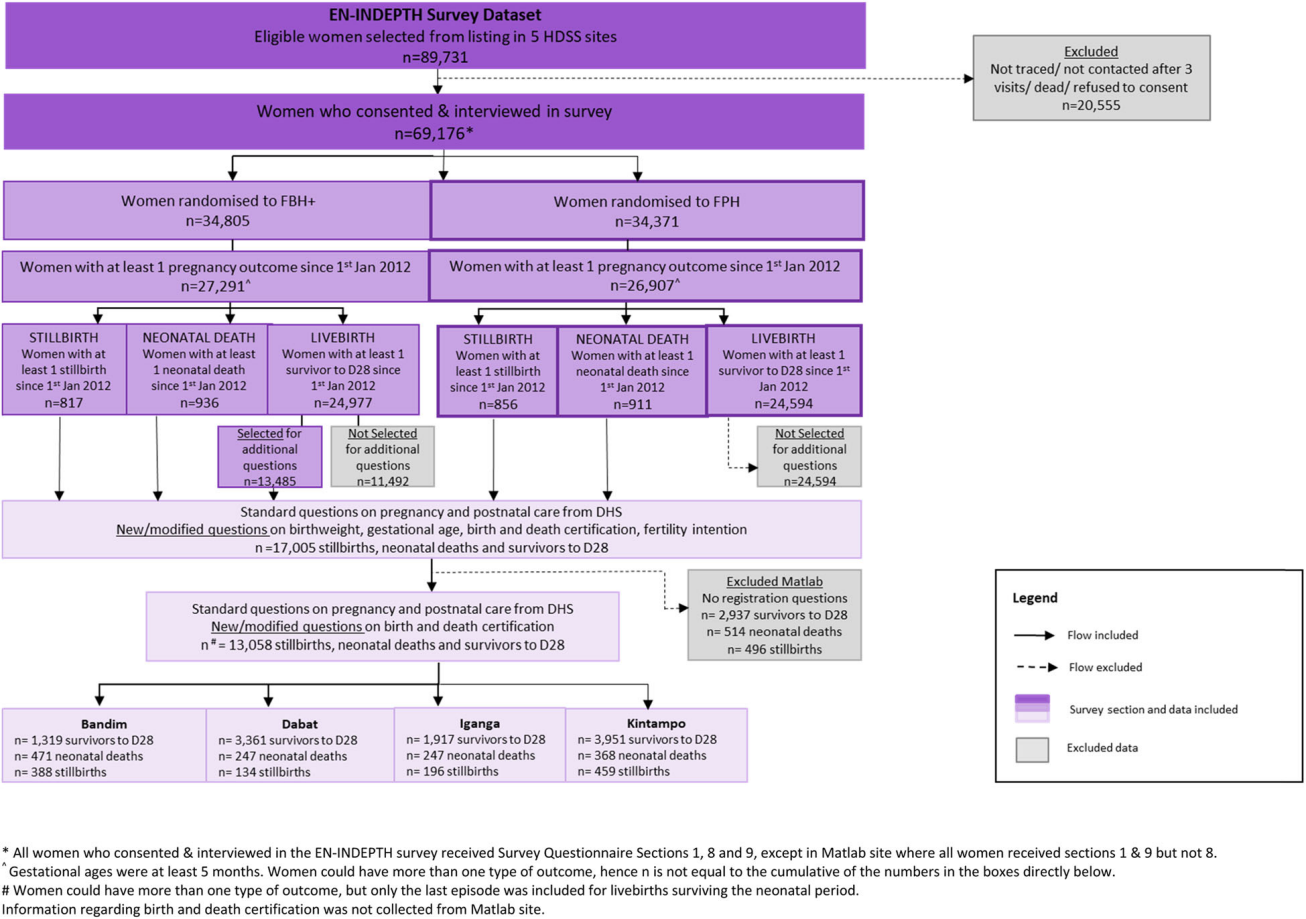
Flow diagram of EN-INDEPTH study population showing data included for birth, stillbirth and death registration

Information on birth and death registration was collected for 13,058 babies and children born to 12,462 surveyed women: Bandim (2065 women), Dabat (3606), Iganga (2254) and Kintampo (4537). These included 1177 stillborn and 11,881 liveborn babies, of whom 1333 died in the neonatal period and 10,548 survived to the 28th day of life (Fig. 1). Survey respondents differed across HDSS sites with regards to age, parity, education and place of birth. Most births took place in health facilities (63.3%); however, in Dabat HDSS, more than half (57.9%) of the births took place outside a health facility (Additional file 4).

### Objective 1: Evaluate survey question performance

Responses to birth and death registration questions were recorded for all women.

A response of ‘don’t know’ for ‘*Does (name) have a birth certificate?*’ was low overall (1.6% of responses for children surviving the neonatal period, 1.9% for neonatal deaths and 3.7% for stillbirths) (Tables 3 and 4B). The additional probe question ‘*Has (name)'s birth been registered with the civil authorities*?’ resulted in a 4-percentage point increase in the estimated proportion of babies registered for children surviving the neonatal period and 0.5-percentage point increase for stillbirths and neonatal deaths (Tables 3 and 4B). However, the increase varied by site, with a minimal effect in Bandim and Kintampo, but resulting in a more than two thirds increase in the number of children surviving the neonatal period reported to have been registered in Dabat and IgangaMayuge.

**Table 3:** Summary of birth registration question responses for livebirths by child’s sex and HDSS site

	**Number of live births (n)**	**Registration with civil authorities (%)**				**% registered with civil authorities**	**% of children registered for whom time from birth to registration was reported**
		Registered and have birth certificate	Registered but don't have birth certificate	Don't know whether registered	Didn't register		
***Children surviving the neonatal period***							
Overall	10,548	26.7	4.0	1.6	67.7	30.7	93.6
**Child sex**							
Female	5,319	26.3	4.3	1.8	67.6	30.7	92.9
Male	5,229	27.0	3.7	1.5	67.8	30.6	94.3
**HDSS site**							
Bandim	1,319	22.0	0.9	0.8	76.3	22.9	93.7
Dabat	3,361	3.7	2.4	3.6	90.3	6.1	73.1
IgangaMayuge	1,917	17.6	14.4	0.9	67.1	31.9	94.1
Kintampo	3,951	52.2	1.3	0.5	46.0	53.5	95.4
***Neonatal deaths***							
Overall	1,333	1.3	0.5	1.9	96.3	1.7	82.6
**Child sex**							
Female	519	1.0	0.0	2.3	96.7	1	62.5
Male	814	1.1	0.7	1.6	96.6	2.9	93.3
**HDSS site**							
Bandim	471	0.4	0.0	0.4	99.2	0.4	50
Dabat	247	1.2	0.0	5.7	93.1	1.2	100
IgangaMayuge	247	3.6	2.0	2.0	92.4	5.7	78.6
Kintampo	368	0.8	0.3	1.1	97.8	1.1	100

Most women were able to provide details of the time since birth to registration for their baby (82.6% of neonatal deaths and 93.6% of children surviving the neonatal period) (Table 3). Response to this question was similar for male and female babies, but varied by HDSS sites, with over 90% reporting in three sites, and compared to fewer than two thirds in Dabat (*p* < 0.001).

Most women (> 95%) reported that they had not registered the death of their baby, with 1.2% of women with a neonatal death and 1.8% with a stillbirth responding that they did not know if the baby’s stillbirth or death had been registered (Tables 4A and 4B).

**Table 4A:** Summary of neonatal death registration question responses by child’s sex and HDSS site

	Number of deaths, *n*	Death registered, %	Don’t know if death registered, %	Not registered, %	Missing, %
**Overall**	1333	1.2	1.2	97.4	0.2
**Reported Birth registered**					
Yes	23	21.7	4.3	73.9	0.0
No	1288	0.9	1.1	98.1	0.0
Not known	19	0.0	5.3	94.7	0.0
Missing	3	0.0	0.0	0.0	100
**Child sex**					
Female	519	1.2	1.2	97.3	0.1
Male	814	1.2	1.2	97.4	0.4
**HDSS site**					
Bandim	471	1.5	1.1	96.8	0.6
Dabat	247	1.2	3.2	95.6	0.0
IgangaMayuge	247	1.2	1.2	97.6	0.0
Kintampo	368	0.8	0.0	99.2	0.0

**Table 4B:** Summary of stillbirth registration question responses by HDSS site

	Overall number of stillbirths	Responses to questions on birth registration for stillbirths					Responses to questions on death registration for stillbirths				Overall
		Reported having a birth certificate (%)	No birth certificate, but registered with civil authorities (%)	Total registered (birth certificate or registered with civil authorities) (%)	Reported don’t know to both birth registration questions (%)	Total registered for whom time from birth to registration was reported (%)	Reported death registered (%)	Reported don’t know if death registered (%)	Reported death not registered (%)	Missing data on death registration status (%)	Total reported as registered^a^ (%)
**Overall**	1177	1.2	0.5	1.7	3.7	1.5	1.1	1.8	96.3	0.9	2.5
**HDSS site**											
Bandim	388	0	0	0	1.0	0	1.3	1.8	94.6	2.3	1.3
Dabat	134	0.8	0	0.8	3.0	0.7	0.7	3.0	96.3	0	0.7
Iganga Mayuge	196	4.1	3.1	7.1	4.1	6.6	3.1	1.5	94.9	0.5	9.2
Kintampo	459	1.1	0	1.1	5.9	0.9	0.2	1.5	98.3	0	1.1

^a^Stillbirths reported as registered on either the birth or death registration questions

Reported median time from birth to registration did not vary by child’s sex but did by site from just over 7.5 months in Dabat and to 1 month in IgangaMayuge. The few neonatal deaths that had their births registered were registered sooner after birth than children surviving the neonatal period (median time from birth to registration: neonatal deaths 1 month (Interquartile range (IQR), 0–3)), children surviving the neonatal period 3 months (IQR, 1–6) (Additional file 4). There was some evidence of heaping at 6-monthly intervals in all sites, which was most marked at 12 months in three sites and at 18 months in Kintampo (Additional file 4).

The mean time to complete birth and death registration questions was less than 1 min in all sites, with 99% of respondents from each of the four sites taking less than 5 min (Additional file 4).

### Objective 2: Assess utility of survey data

#### Data on completeness of registration

**Fig. 2:**
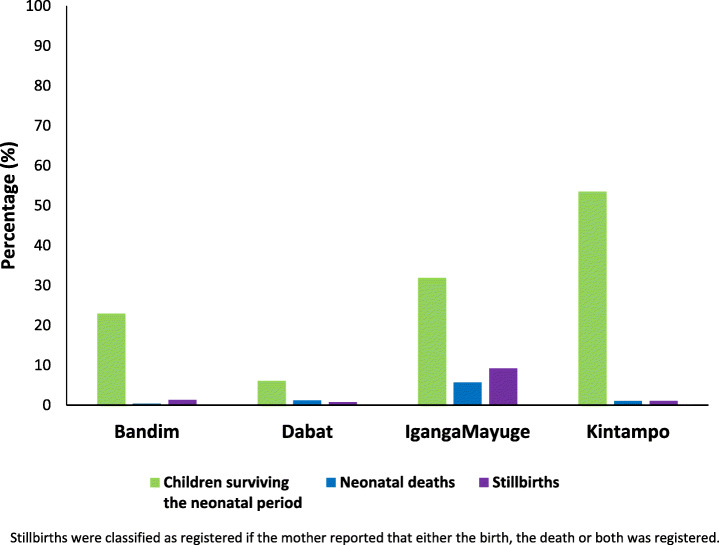
Women’s report of completeness of birth registration in EN-INDEPTH survey by outcome (*n* = 10,548)

Overall, 30.7% of children surviving the neonatal period and 1.7% of neonatal deaths were reported to have had their births registered with the civil authorities. For children surviving the neonatal period, completeness of birth registration was the highest in Kintampo (53.5%), compared to 6.1% in Dabat (Fig. 2). Reported completeness was similar to nationally reported completeness from recent surveys (Table 5). Reported completeness of birth registration for neonatal deaths in all sites was very low compared to completeness for children surviving the neonatal period (*p* < 0.001).

**Table 5:** Completeness of birth registration for children surviving the neonatal period in EN-INDEPTH study compared to external data sources (*n* = 10,548)

HDSS name	Birth registration completeness reported in EN-INDEPTH survey using single question (2012–2018)	Birth registration completeness reported in EN-INDEPTH survey using two questions (2012–2018)	Birth registration completeness in DHS/MICS survey (national)^**a**^
**Bandim (Guinea-Bissau)**	290 (22.0%)	302 (22.9%)	24% MICS 2014
**Dabat (Ethiopia)**	124 (3.7%)	205 (6.1%)	3% DHS 2016
**IgangaMayuge (Uganda)**	337 (17.6%)	612 (31.9%)	32% DHS 2016
**Kintampo (Ghana)**	2063 (52.2%)	2114 (53.5%)	71% DHS 2014

^**a**^Data source: data.unicef.org

Estimates from the EN-INDEPTH include only children alive after the neonatal period. This will slightly overestimate population-based coverage due to very low coverage of birth certification for neonatal deaths. Estimates from standard DHS only include coverage for currently alive children in the roster, which may overestimate further coverage as child deaths at any age would be excluded.

Fewer than 2% of neonatal deaths had their deaths registered in all sites (Table 4A, Additional file 4). 9.2% of stillbirths were reported by their mothers to have been registered in IgangaMayuge, with fewer than 1.5% in other sites (Table 4B, Additional file 4).

#### Age at registration

**Fig. 3:**
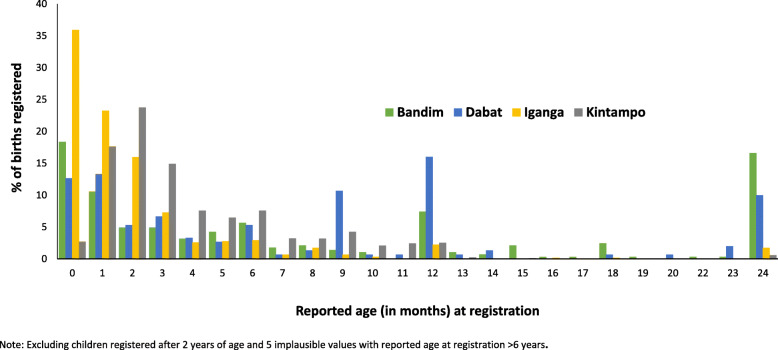
Age at registration for children surviving the neonatal period registered by age 24 months (*n* = 2972)

The majority of registered surviving babies were registered during the first few months of life, with some evidence of catch up registration especially in the Dabat and Bandim sites (Fig. 3).

#### Factors associated with non-registration

For children surviving the neonatal period, being under 1 year of age at the time of the survey, born at home and maternal socio-demographic factors including younger age, higher parity, lower levels or no education and lower socio-economic status were associated with non-registration in both crude and adjusted analyses. Non-registration was not associated with the sex of the child (Additional file 4).

#### Reasons for non-registration

**Fig. 4:**
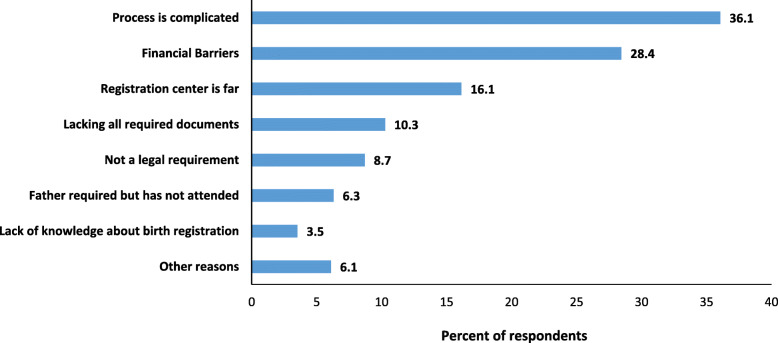
Reasons for non-registration for children surviving the neonatal period (*n* = 7312)

7,312 out of 10,548 (69.3%) of women with a non-registered child surviving the neonatal period provided information on barriers to registration. Amongst these, 36.1% of women reported that the birth registration process was too complicated to understand. Other commonly reported barriers included cost (28.4% of respondents) and distance to a registration facility (16.1%). Lack of the required documentation, father’s support and a name for the baby were less frequently reported as barriers (Fig. 4, Additional file 4).

#### Gap analysis

**Fig. 5:**
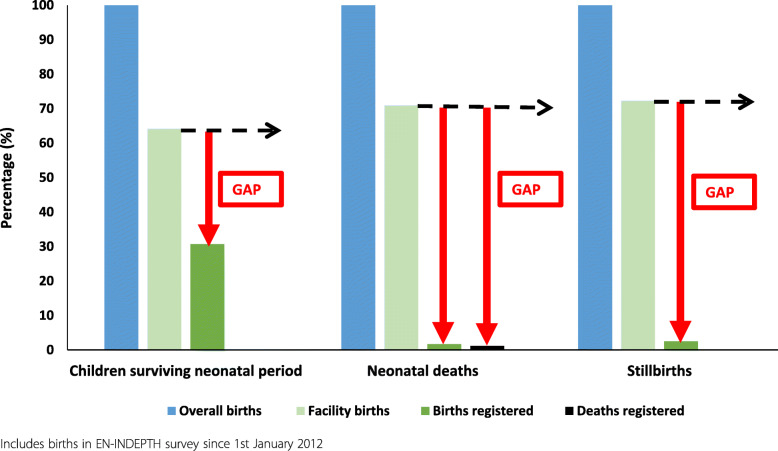
Gap analysis for birth, stillbirth and neonatal death registration, EN-INDEPTH survey (*n* = 13,058)

Half of surviving babies that were born in a facility were reported to have had their births registered by the time of the survey; however, large between-site variation was observed (Fig. 5, Additional file 5). Kintampo has nearly closed the facility birth registration gap for children surviving the neonatal period with 81% of facility births registered, compared to 35% in IgangaMayuge, 31% in Bandim and just 14% in Dabat. In comparison, just 2.4% of neonatal deaths and 3.5% of stillbirths who were born in a facility were registered with the reported registration gap consistently large across all sites.

## Discussion

Household surveys are an important source of population-level information on completeness of birth registration, and our study involved 13,058 births in four countries with varying contexts for registration. To our knowledge, this is the first study to assess the completeness and quality of data from existing survey questions on birth registration, and importantly to also evaluate new questions regarding registration of stillbirths and neonatal deaths. We also assessed how long the birth registration questions took to answer and explored factors associated with non-registration.

For birth registration, we found that women were able to answer, with no missing data and fewer than 5% don’t know responses across all the questions asked. The time to administer these questions was short, with the full set of birth registration questions taking on average less than 1 min to complete in all sites.

Birth registration reported completeness was low at 30.7% overall for babies surviving the neonatal period (with variation across the four study sites, being lowest in Dabat, Ethiopia), compared to birth registration for just 1.7% of neonatal deaths. Lower completeness of birth and death registration was expected in Dabat because Ethiopia’s first permanent, compulsory and universal registration and certification system for vital events throughout the country is very new, only launched in August 2016 (Table 1). Therefore, national civil registration was still in the early stages during the study period [18]. Higher rates of completeness would be expected in other sites, especially in Kintampo, where an active programme was underway to increase birth registration from 2010 to 2014. For almost all registered children surviving the neonatal period, women were able to report age at registration with a plausible distribution apart from some heaping at 6-month intervals. The majority of children whose births were registered in the first 2 years of life were reported to have been registered during the first 6 months of life. In our study, very few children were registered after 2 years of age. This finding is in contrast to other studies which have found a peak in birth registrations around 5 or 6 years of age, especially where required for school entry [3, 19]. However, as the median age of the children in this study was only 25 months, our study was not designed to detect later registration peaks. Similar to previous studies, lack of birth registration was associated with home birth, lower socio-economic and educational status, but not with the sex of the child [3, 20–24].

Barriers to birth registration are asked in the MICS for 45 countries and in half of these countries, carers reported not knowing how to register the child (Table 2). However in the remaining countries, most caregivers of unregistered children seem aware of the birth registration process [3]. Our study provided more detail by asking new questions to mothers of unregistered children to elucidate if any of seven different potential reasons contributed to non-registration and found complexity of the registration process (36%), cost (28%) and distance to registration facility (16%) as the commonest reported barriers. Our findings regarding knowledge of the registration process and distance are similar to a previous study in Niger [25]; however, more women in our study reported cost to be a barrier. In a previous study in urban Bandim, 42% of women reported lack of pre-requisite documents and 28% the father’s absence as barriers to birth registration; these were less commonly reported as barriers in our multi-site study [11].

Whilst many LMICs are now working on strengthening CRVS, there are major variations between even these four countries (Table 1) [11]. In Uganda, Guinea-Bissau and Ethiopia, legislation regarding mandatory registration of births and deaths exists. However, enforcement of these laws is highly variable. Variation in the period within which to report occurrence of births and enforcement of regulation is one of the limiting factors for complete CRVS. In Guinea-Bissau, a birth is by law reported within 30 days, 90 days in Ethiopia and anytime in Uganda. Enforcing penalties on late registration of births is reported as a challenge in some countries such as Ethiopia and Ghana and where no penalties are legislated, like in Uganda, there are still reporting, registration and certification challenges of vital statistics [26].

Importantly, stillbirth and death registration is not included in either DHS or MICS standard questionnaires. Completeness of stillbirth and death registration has lagged behind birth registration, and whilst completeness has been assumed to be low, no previous estimates of completeness have been made using survey data [2] and indeed very few population-based studies have assessed completeness of stillbirth or death registration [27]. Our new questions were asked for 2510 stillbirths and neonatal deaths and were found to have high response rates.

We found a shockingly large gap for stillbirth registration. Currently, of the four countries included in this study, only Ghana has a legal provision for the registration of stillbirths (Table 1). Despite 72.9% of reported stillbirths occurring in facilities, only around 1% were reported to be registered in three sites, with 9.2% reported being registered in IgangaMayuge. In view of the lack of requirement for registration of stillbirths or a formal stillbirth or fetal death register in Guinea-Bissau, Ethiopia or Uganda, it is possible that women may have misunderstood the registration questions and reported ‘registration’ within the HDSS, e.g. when the pregnancy was ‘registered’, or some women may have misreported neonatal deaths (which could have been registered with the civil authority) as stillbirths, as misclassification between these events in surveys is relatively common [28, 29]. Whilst it is possible in Ghana that some of these stillbirths may have been notified directly to the civil registrar by the health providers for inclusion in the stillbirth or fetal death register as recommended by the United Nations Statistical Division [30], generally completeness of vital statistics for stillbirths in most LMICs is currently very low [31]. A revision of the laws in countries without provision for stillbirth registration is needed to require reporting of late gestation stillbirths, as a minimum, and investment in training and support to implement this legislation in accordance with United Nations guidelines [30]. Enabling registration of all facility stillbirths, with information on timing (antepartum/ intrapartum) and cause of death where feasible using the WHO Medical Certificate for Cause of Death, would greatly increase the availability of data to improve stillbirth estimates and tracking of progress towards ending preventable stillbirths [13, 32]. Once these changes are in place, measuring completeness of stillbirth registration will require design and testing of survey questions that are stillbirth-specific.

The reported completeness of neonatal death registration in this study was even lower than for stillbirths (1.2%), and consistent with the World Health Organization's estimate that fewer than 5% of neonatal deaths globally are registered [33]. A study undertaken in the urban Bandim site found that reported completeness of birth registration for neonatal deaths was much lower than for children surviving the neonatal period [11]. This presents a large gap in vital statistics for these babies which could be partly closed by improving facility-based notification of all births and mandating that both birth and death must be notified and recorded in the case of a neonatal death. Notifying every birth at the time of birth and building strong linkages between civil registration systems and health programmes could enable health programmes to identify live births eligible for services such as postnatal care and immunisation and to follow-up defaulters to identify children who have died and enable provision of care for surviving children. The introduction of local mechanisms for community health staff to serve as notifiers of stillbirths, neonatal and infant deaths could improve capture of these events when they occur outside facilities, as families have little incentive to register them.

Closing the gap for registration of facility births and also deaths around the time of birth, notably stillbirths, could address common reasons for non-registration and lead to large increases in completeness of birth, stillbirth and neonatal death registration in all sites. This is feasible to achieve. UNICEF, WHO and the Global Vaccine Alliance (Gavi) have provided successful examples for integrating CRVS and health systems, in particular immunisation systems, in recent reports including creating awareness of the importance of registration during antenatal and delivery care; ensuring all births and deaths occurring in health facilities are notified to the civil registrar, with death notification including cause of death; increasing the potential co-location of registration facilities within hospitals and other delivery facilities; notifying home births and deaths by community health workers; notifying unregistered children when presented for immunisation and other health services; promoting community outreach for creating demand for birth and death registration; and sensitising health workers on the importance of registration of births and deaths [3, 34–36]. However, if these strategies are to be successful, frontline health workers, managers and other stakeholders must be included in the design and roll out of systems to link health management information systems and CRVS [37]. In addition, further investment, training and resources are required to improve the classification and reporting of stillbirths and early neonatal deaths. These are required to reduce misclassification and ensure that comparable information is recorded for all these deaths in the vital statistics system, for example through health providers notifying all these events through a common notification system.

This study has strengths, notably being undertaken across four different settings in sub-Saharan Africa, including information on a large number of children (including 2510 stillbirths and neonatal deaths). However, we note that whilst women were able to provide plausible responses to these questions, we were unable to verify accuracy by comparing responses to actual birth or death registration records. Since this study was undertaken in HDSS sites amongst populations under surveillance, it is possible that this may affect women’s responses, although in none of these sites are women routinely asked about birth or death registration of their children. Potential confusion between ‘registration’ with the HDSS, religious institutions or other groups and registration with the civil authorities, as highlighted in a UNICEF report [19], may have occurred, particularly with respect to stillbirth and death registration. The completeness of birth and death registration may therefore be even lower than we have estimated.

Reliable measures in surveys are crucial to track progress for birth and death registration. Surveys have the advantage of providing data that can be disaggregated by different categories such as place of residence, sex, maternal education, or socio-economic status to identify which children are being left behind. This study found that questions on both birth and death registration were feasible to ask in a household survey, with minimal additional time implications. Whilst asking birth registration questions for surviving children in the household or the child’s questionnaire has the advantage of capturing information on all children regardless of whether the mother is in the household or eligible to be interviewed, children who are stillborn or who have died are missed. Asking additional questions on birth registration for non-surviving children in the woman’s questionnaire could provide information on deceased children who may be at higher risk of not being registered.

## Conclusions

Given that around 80% of the world’s births are now in facilities, closing the gap between facility birth and registration for these babies would increase birth and stillbirth registration completeness and enable timely tracking through routine facility data and annual vital statistics reports, instead of relying only on 5-yearly surveys. However, facility registration alone will leave behind 20% of all babies who are born outside facilities, who are often the poorest and at highest risk of stillbirth and neonatal mortality. Therefore, surveys remain crucial to track overall population-level progress for birth registration and to identify who is left behind in this marker for child rights.

There is a larger gap for death registration, with only 1.2% of neonatal deaths and 2.5% of stillbirths reported to be registered. More attention is required to capture these deaths in facility and community systems, count them through death certificates and improve cause of death data to count and end these deaths, which will be crucial for all governments if SDG targets are to be measured and met.

## Supplementary information

**Additional file 1.** Background overview of the four HDSS sites.

**Additional file 2.** Selection of women with a livebirth surviving the neonatal period.

**Additional file 3.** STROBE guidelines checklist.

**Additional file 4.** Additional results.

**Additional file 5.** Gap analysis for birth registration in EN-INDEPTH survey, by site.

**Additional file 6.** Ethical Approval of local Institutional Review Boards.

## Data Availability

Data sharing and transfer agreements were jointly developed and signed by all collaborating partners. The datasets generated during the current study are deposited online at 10.17037/DATA.00001556 with data access subject to approval by collaborating parties.

## Abbreviations

CRVS: Civil registration and vital statistics
DHS: Demographic and health survey
EN-INDEPTH: Every Newborn-International Network for the Demographic Evaluation of Populations and their Health
FBH+: Full birth history (“+” denotes additional questions on pregnancy losses)
FPH: Full pregnancy history
HDSS: Health and demographic surveillance system
LMIC: Low- and middle-income countries
MICS: Multiple Indicator Cluster Survey
SDG: Sustainable Development Goals
