# Supplementary material for: Birth, stillbirth and death registration data completeness, quality and utility in population-based surveys: EN-INDEPTH study
Source: Popul Health Metr. 2021 Feb 8;19(Suppl 1):14. doi: 10.1186/s12963-020-00231-2 (PMC7869445; doi:10.1186/s12963-020-00231-2)
Supplement: Supplementary file 1 — Additional file 1. Background overview of the four HDSS sites. [file 12963_2020_231_MOESM1_ESM.docx]

## Additional file 1: Background overview of the four HDSS sites

|  | **Bandim** | **Dabat** | **IgangaMayuge** | **Kintampo** |
| --- | --- | --- | --- | --- |
| **Country** | Guinea Bissau | Ethiopia | Uganda | Ghana |
| **HDSS start year** | 1978 | 1996 | 2004 | 1994 |
| **Location** | Guinea-Bissau, covering rural and urban | Gondor, Amhara regional state, 821 km northwest of Addis Ababa and 75km north | Iganga and Mayuge districts, approximately, 120km east of capital, Kampala along Kenya-Uganda highway | Brong Ahafo region |
| **Population** | 180,000 | 69,468 | 83,000 | 152,519 |
| **Households** | 36,000 | 16,016 | 16,000 | 32,000 |
| **Total Fertility Rate** | 4.3 | 3.8 | 4.3 | 4.1 |
| **Frequency of rounds** | Urban: Monthly Rural: 2/year | 2/year | 2/year | 1/year |
| **Informants/**  **scouts** | Recently started using community key informants | Local guides report within 48 hours | 64 Community based “scouts”  and VHTs | Community key informants |
| **Incentives for reporting** | Each woman asked pregnancy status and pregnancy ID given to pregnancy | 83% female enumerators | Informants and scouts are community members who are elected and accepted by the communities | Each woman asked pregnancy status |
| **Frequency of re-census the area** | Rural: Every round; Urban; Dependent on funding, every 2-7 years. | Every 7 years | Each update round | Last census 2003 |
| **Facility births** | Urban: 65%  Rural: 39% | 17% | 64% | 61% |
| **Birth certificate** | Yes – On a subset around 400 women | No | No | No |
| **Death certificate** | Yes – asked with verbal autopsy | No | Yes – asked with verbal autopsy | Yes – asked with verbal autopsy |
| **Miscarriages/TOP** | Yes – Likely only miscarriages reported | Yes - TOP differentiated from miscarriages | Yes - Miscarriages only | Yes - TOP differentiated from miscarriages |
