# Supplementary material for: Birth, stillbirth and death registration data completeness, quality and utility in population-based surveys: EN-INDEPTH study
Source: Popul Health Metr. 2021 Feb 8;19(Suppl 1):14. doi: 10.1186/s12963-020-00231-2 (PMC7869445; doi:10.1186/s12963-020-00231-2)
Supplement: Supplementary file 4 — Additional file 4. Additional results. [file 12963_2020_231_MOESM4_ESM.docx]

## Additional file 4: Additional results

### Additional file 4.1: Characteristics of the births included in the EN-INDEPTH birth certification analysis for four sites

|  | **Bandim** | **Dabat** | **IgangaMayuge** | **Kintampo** | **Total** |
| --- | --- | --- | --- | --- | --- |
|  | n(%) | n(%) | n(%) | n(%) | n(%) |
| **Child's characteristics** | | | | | |
| **Sex** | | | | | |
| Male | 1053(48.4) | 1831(48.9) | 1128(47.8) | 2320(48.6) | 6332(48.5) |
| Female | 902(41.4) | 1823(48.7) | 1125(47.7) | 2177(45.6) | 6027(46.2) |
| Unknown^1^ | 223(10.2) | 88(2.4) | 107(4.5) | 281(5.9) | 699(5.4) |
| **Child's vital status** |  |  |  |  |  |
| Stillbirths | 388(17.8) | 134(3.6) | 196(8.3) | 459(9.6) | 1177(9.0) |
| Neonatal deaths | 471(21.6) | 247(6.6) | 247(10.5) | 368(7.7) | 1333(10.2) |
| Survived the neonatal period | 1319(60.6) | 3361(89.8) | 1917(81.2) | 3951(82.7) | 10548(80.8) |
| *Median age (months) at time of survey (IQR)^2^* | 22 (10 – 38) | 25 (12 – 42) | 21 (10 – 38) | 26 (15 – 41) | 25 (12 – 41) |
| **Total** | **2,178** | **3,742** | **2,360** | **4,778** | **13,058** |
|  |  |  |  |  |  |
| **Mother characteristics** | | | | | |
| **Age of the mother** | | | | | |
| 15-19 | 118(5.7) | 191(5.3) | 182(8.1) | 164(3.6) | 655(5) |
| 20-24 | 512(24.8) | 631(17.5) | 490(22) | 710(16) | 2343(19) |
| 25-29 | 515(24.9) | 914(25.4) | 489(22) | 983(22) | 2901(23) |
| 30-34 | 476(23.1) | 721(20) | 470(21) | 1056(23) | 2723(22) |
| 35+ | 444(21.5) | 1149(31.9) | 623(28) | 1624(36) | 3840(31) |
| **Education level** | | | | | |
| No education | 655(31.7) | 2238(62.1) | 214(9.5) | 1840(40.6) | 4947(39.7) |
| Primary Only | 560(27.1) | 802(22.2) | 1295(57.5) | 1898(41.8) | 4555(36.6) |
| Primary & Secondary | 691(33.5) | 271(7.5) | 659(29.2) | 770(17.0) | 2391(19.2) |
| Higher | 159(7.7) | 295(8.2) | 86(3.8) | 29(0.6) | 569(4.6) |
| **Religion** | | | | | |
| Christian | 765(37.1) | 3484(96.6) | 926(41.1) | 2793(62) | 7968(64) |
| Muslim | 865(41.9) | 122(3.4) | 1326(58.8) | 1492(33) | 3805(31) |
| Other | 200(9.7) | 0 | 2(0.1) | 53(1) | 255(2) |
| None | 235(11.4) | 0 | 0 | 199(4) | 434(3) |
| **Birth place** | | | | | |
| Health facility | 1,480(71.7) | 1,518(42.1) | 1,935(85.9) | 2,955(65.1) | 7,888(63.3) |
| Others | 585(28.3) | 2,088(57.9) | 319(14.2) | 1,582(34.9) | 4,574(36.7) |
| **Wealth quintile** | | | | | |
| 1 | 417(20.2) | 943(26.2) | 695(30.8) | 1021(23) | 3076(24.7) |
| 2 | 411(19.9) | 679(18.8) | 561(24.9) | 911(20) | 2562(20.6) |
| 3 | 382(18.5) | 757(21) | 435(19.3) | 836(18) | 2410(19.3) |
| 4 | 400(19.4) | 680(18.9) | 334(14.8) | 848(19) | 2262(18.2) |
| 5 | 455(22) | 547(15.2) | 229(10.2) | 921(20.3) | 2152(17.3) |
| **Parity^3^** | | | | | |
| 0 | 1(0.1) | 5(0.1) | 4(0.2) | 1(0.02) | 11(0.1) |
| 1 | 458(22.2) | 593(16.4) | 351(15.6) | 670(14.8) | 2072(16.6) |
| 2 | 500(24.2) | 595(16.5) | 342(15.2) | 783(17.3) | 2220(17.8) |
| 3 | 368(17.8) | 499(13.8) | 260(11.5) | 766(16.9) | 1893(15.2) |
| 4 | 256(12.4) | 491(13.6) | 260(11.5) | 684(15.1) | 1691(13.6) |
| 5+ | 482(23.3) | 1423(39.5) | 1037(46) | 1633(36) | 4575(36.7) |
| **Total^4^** | **2,065** | **3,606** | **2,254** | **4,537** | **12,462** |

^1^ No information was available on sex of the child for stillbirths in the FBH arm

^2^ IQR=interquartile range

^3^ Parity refers to the total number of pregnancies that a woman has carried to age of viability (including live and stillbirths)

^3^ Number of births is not equal to the number of women as some women reported birth and death certification status for more than one type of birth e.g. stillbirth, neonatal death, a child surviving the neonatal period

###

### Additional file 4.2: Median time since birth to birth registration in months for registered children by outcomes (n=3,063)

|  | Overall Number of babies | Registered births reporting time from birth to registration  N (%) | Number of implausible values of time since birth to registration^1^ | Median time since birth in months to registration  (IQR) |
| --- | --- | --- | --- | --- |
| *Children surviving the neonatal period* |  |  |  |  |
| Overall | 10,548 | 3,026(28.7) | 10 | 3(1-6) |
| Child sex |  |  |  |  |
| Female | 5,319 | 1,510(28.4) | 4 | 3(1-6) |
| Male | 5,229 | 1,516(29.0) | 6 | 3(1-6) |
| HDSS site |  |  |  |  |
| Bandim | 1,319 | 283(21.5) | 1 | 6(1-24) |
| Dabat | 3,361 | 150(4.5) | 0 | 7.5(1-12) |
| IgangaMayuge | 1,917 | 576(30.0) | 3 | 1(0-2) |
| Kintampo | 3,951 | 2,017(51.1) | 6 | 3(2-6) |
|  |  |  |  |  |
| *Neonatal deaths* |  |  |  |  |
| Overall | 1333 | 19(1.4) | 1 | 0.5(0-3) |
| Child sex |  |  |  |  |
| Female | 519 | 5(0.96) | 1 | 0.5(0-1.5) |
| Male | 814 | 14(1.72) | 0 | 0.5(0-3) |
| HDSS site |  |  |  |  |
| Bandim | 471 | 1(0.2) | 1 | . |
| Dabat | 247 | 3(1.2) | 0 | 5(0-10) |
| IgangaMayuge | 247 | 11(4.5) | 0 | 0(0-2) |
| Kintampo | 368 | 4(1.1) | 0 | 1(0.5-3) |
|  |  |  |  |  |
| Stillbirths |  |  |  |  |
| Overall | 1,177 | 18(1.5) | 0 | 1.5(0-7) |
| HDSS site |  |  |  |  |
| Bandim | 388 | 0 | 0 | . |
| Dabat | 134 | 1(0.7) | 0 | 12(12-12) |
| IgangaMayuge | 196 | 13(6.6) | 0 | 1(0-3) |
| Kintampo | 459 | 4(0.9) | 0 | 5(2-8) |

^1^ An age at registration was categorised as implausible if the state age at registration was greater than the current age of the child at the time of the survey

### Additional file 4.3A: Box plot showing median age in months by child’s sex

### Additional file 4.3B: Box plot showing median age in months by HDSS

### Additional file 4.4: Heaping of reported age at registration in months for babies surviving the neonatal period

Age at registration in months was asked for babies registered before 24 months of age. Heaping indices were calculated to investigate potential heaping at 6, 9, 12 and 18 months. Heaping index calculated as: (Number of babies registered in a given month/ Average number registered by month for given age +/- 2 months). It was not possible to calculate a heaping index for 24 months as no data was capture on registrations at 24 – 26 months.

Table: Heaping index for reported age at registration in months in children surviving the neonatal period registered by age 24 months

|  | **Bandim**  **(Guinea-Bissau)** | **Dabat**  **(Ethiopia)** | **IgangaMayuge**  **(Uganda)** | **Kintampo**  **(Ghana)** |
| --- | --- | --- | --- | --- |
| Registrations 6 months | 16 | 8 | 17 | 153 |
| Registrations at 4 – 8 months^1^ | 48(9.6) | 20(4) | 62(12.4) | 566(113.2) |
| **Heaping index 6 months** | **1.7** | **2** | **1.4** | **1.4** |
| Registrations 9 months | 4 | 16 | 4 | 86 |
| Registrations at 7 – 11 months^1^ | 18(3.6) | 21(4.2) | 20(4) | 306(61.2) |
| **Heaping index 9 months** | **1.1** | **3.8** | **1** | **1.4** |
| Registrations 12 months | 21 | 24 | 13 | 51 |
| Registrations at 10-14 months^1^ | 29(5.8) | 29(5.8) | 15(3) | 147(29.4) |
| **Heaping index 12 months** | **3.6** | **4.1** | **4.3** | **1.7** |
| Registrations 18 months | 7 | 1 | 1 | 1 |
| Registrations at 16-20 months^1^ | 10(2) | 2(0.4) | 2(0.4) | 1(0.2) |
| **Heaping index 18 months** | **3.5** | **2.5** | **2.5** | **5** |

^1^ number of registrations over the period shown with average registrations per month in brackets

### Additional file 4.5: Time taken to answer questions on birth registration

| Time taken(0-30min) | Bandim | Dabat | IgangaMayuge | Kintampo | Total |
| --- | --- | --- | --- | --- | --- |
|  |  |  |  |  |  |
| <1 | 1,321 | 3,546 | 2,003 | 4,283 | 11,153 |
| 1-<2 | 211 | 156 | 186 | 366 | 919 |
| 2-<3 | 54 | 28 | 40 | 91 | 213 |
| 3-<4 | 11 | 8 | 14 | 30 | 63 |
| 4-<5 | 2 | 6 | 11 | 8 | 27 |
| 5-<6 | 1 | 0 | 8 | 5 | 14 |
| 6-<7 | 0 | 1 | 1 | 3 | 5 |
| 7-<8 | 0 | 0 | 0 | 1 | 1 |
| 8-<9 | 0 | 1 | 1 | 0 | 2 |
| 9-<10 | 2 | 1 | 0 | 2 | 5 |
| 10-<11 | 0 | 0 | 0 | 0 | 0 |
| 11-<12 | 0 | 0 | 1 | 1 | 2 |
| 12-<13 | 0 | 0 | 1 | 1 | 2 |
| 13-<16 | 0 | 0 | 0 | 1 | 1 |
| 16-<17 | 0 | 0 | 2 | 0 | 2 |
| 17-<18 | 0 | 0 | 1 | 1 | 2 |
| 18-<20 | 0 | 0 | 3 | 0 | 3 |
| 20-<23 | 0 | 0 | 0 | 1 | 1 |
| ≥23 | 0 | 0 | 0 | 1 | 1 |
|  |  |  |  |  |  |
| Total | 1,602 | 3,747 | 2,272 | 4,795 | 12,416 |

Numbers exclude 195 records with data system errors

### Additional file 4.6: Women’s report of completeness of registration for neonatal deaths (n=1,333)

**
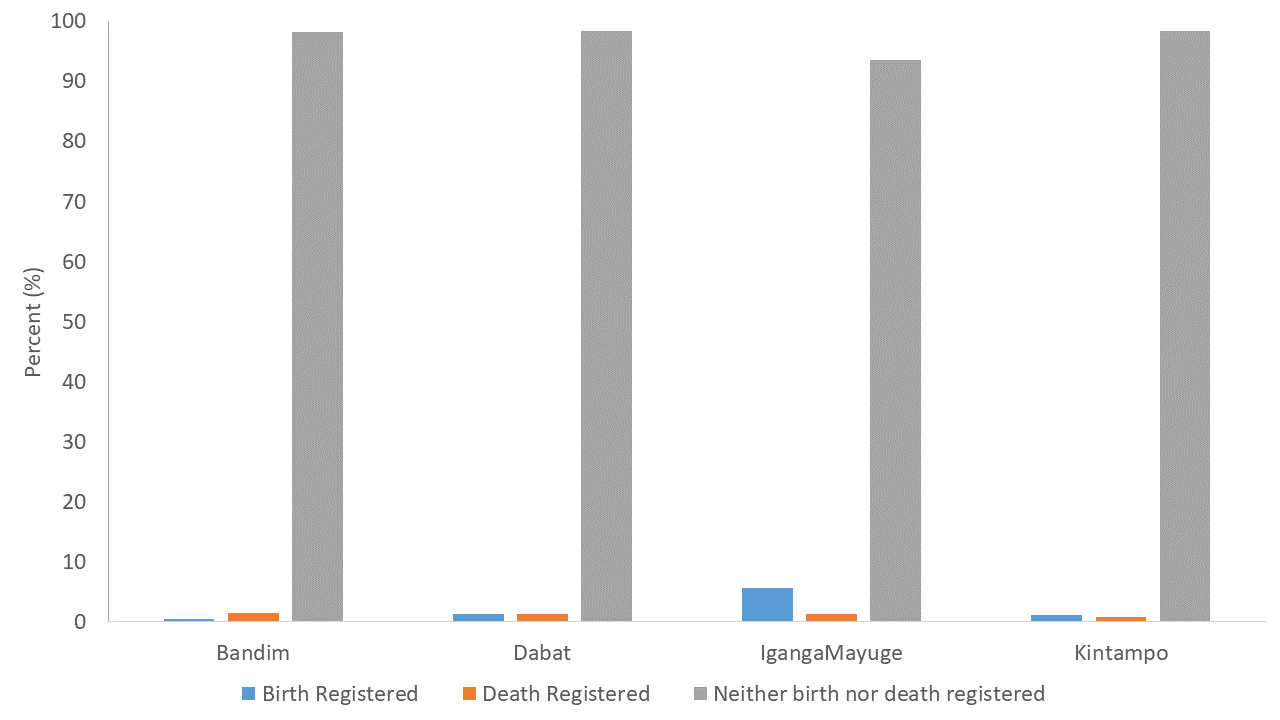
**

### Additional file 4.7: Women’s report of completeness of registration for stillbirths (n=1,177)


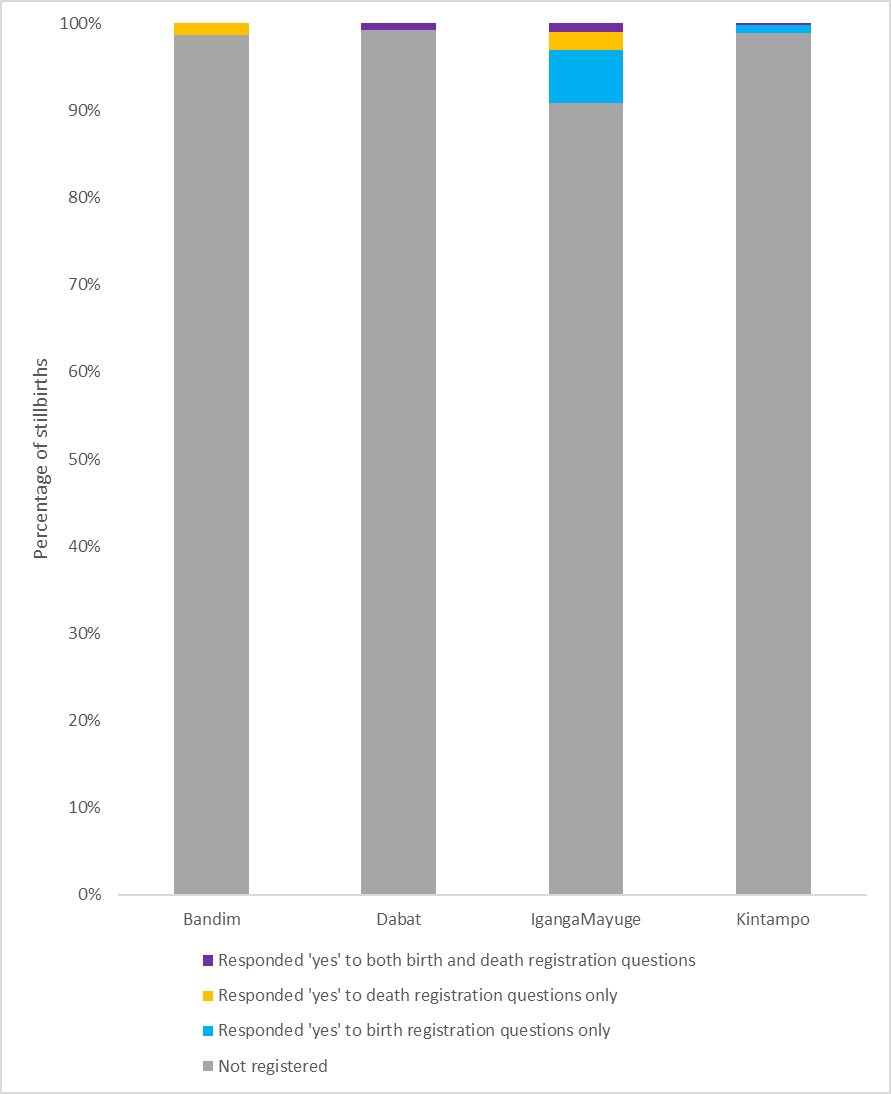


Stillbirths were classified as not registered if mothers responded ‘no’ to all questions regarding birth and death registration with the civil authorities. Patterns of responses varied by site with no women responding yes to birth registration questions for stillbirths in Bandim, and 1.3% reporting the stillbirth was registered as a death (n=5). In Dabat, only 1 stillbirth was classified as registered and the baby’s mother responded ‘yes’ to both birth and death registration questions. In Kintampo, of the 5 stillborn babies classified as registered, all 5 mothers responded ‘yes’ to questions about birth registration and only 1 to the death registration questions. In Iganga, overall 18 (9.2%) of stillbirths were classified as registered – in 12 cases the mother had responded ‘yes’ to the birth registration questions only, in 2 cases she responded ‘yes’ to both the birth and death questions and in 4 cases ‘yes’ to the death registration questions only.

### Additional file 4.8: Age at registration in years for children surviving the neonatal period by HDSS (n=3,021)

Excluding 5 implausible values with reported age at registration >6 years. Includes 3,021 children born since 1^st^ January 2012 who survived the neonatal period. Age at time of survey varied from 1 month to 6 years. Median age at time of survey varied by site from 21 to 26 months (see Table 4.9 below).

### Additional file 4.9: Woman and child characteristics for non-registered children surviving the neonatal period (n=7,312)

|  | **Bandim** | **Dabat** | **IgangaMayuge** | **Kintampo** | **Total** |
| --- | --- | --- | --- | --- | --- |
| **Child's sex** |  |  |  |  |  |
| Boy | 505(49.8) | 1549(49.1) | 652(50) | 913(49.7) | 3619(49.5) |
| Girl | 510(50.2) | 1606(50.9) | 653(50) | 924(50.3) | 3693(50.5) |
| **Child’s age at time of survey** |  |  |  |  |  |
| Median age (months(IQR))^1^ | 22 (10 – 38) | 25 (12 – 42) | 21 (10 – 38) | 26 (15 – 41) | 25 (12 – 41) |
| **Age of the woman** | | | | | |
| 15-19 | 71(7.0) | 167(5.3) | 110(8.4) | 84(4.6) | 432(5.9) |
| 20-24 | 285(28.1) | 550(17.4) | 287(22.0) | 303(16.5) | 1425(19.5) |
| 25-29 | 265(26.1) | 802(25.4) | 288(22.1) | 379(20.6) | 1734(23.7) |
| 30-34 | 191(18.8) | 624(19.8) | 284(21.8) | 410(22.3) | 1509(20.6) |
| 35+ | 203(20.0) | 1012(32.1) | 336(25.7) | 661(36.0) | 2212(30.3) |
| **Education** | | | | | |
| No education | 333(32.8) | 1989(63.0) | 139(10.7) | 872(47.5) | 3333(45.6) |
| Primary only | 296(29.2) | 694(22) | 742(56.9) | 716(39.0) | 2448(33.5) |
| Primary & Secondary | 345(34.0) | 229(7.3) | 373(28.6) | 245(13.3) | 1192(16.3) |
| Higher | 41(4.0) | 243(7.7) | 51(3.9) | 4(0.2) | 339(4.6) |
| **Religion** | | | | | |
| Christian | 354(34.9) | 3053(96.8) | 541(41.5) | 1208(65.8) | 5156(70.5) |
| Muslim | 432(42.6) | 102(3.2) | 764(58.5) | 467(25.4) | 1765(24.1) |
| Other | 99(9.8) | 0 | 0 | 28(1.5) | 127(1.7) |
| None | 130(12.8) | 0 | 0 | 134(7.3) | 264(3.6) |
| **Birth place** | | | | | |
| Health facility | 687(67.7) | 1293(41) | 1116(85.5) | 1015(55.3) | 4111(56.2) |
| Others | 328(32.3) | 1862(59) | 189(14.5) | 822(44.7) | 3201(43.8) |
| **Socio-economic status** | | | | | |
| 1 (Poorest) | 193(19) | 852(27) | 417(32) | 623(33.9) | 2085(28.5) |
| 2 | 191(18.8) | 579(18.4) | 341(26.1) | 438(23.8) | 1549(21.2) |
| 3 | 197(19.4) | 671(21.3) | 254(19.5) | 349(19) | 1471(20.1) |
| 4 | 212(20.9) | 592(18.8) | 183(14) | 247(13.4) | 1234(16.9) |
| 5 (Richest) | 222(21.9) | 461(14.6) | 110(8.4) | 180(9.8) | 973(13.3) |
| **Parity** | | | | | |
| 1 | 259(25.5) | 534(16.9) | 229(17.5) | 233(12.7) | 1255(17.2) |
| 2 | 251(24.7) | 507(16.1) | 193(14.8) | 279(15.2) | 1230(16.8) |
| 3 | 176(17.3) | 438(13.9) | 143(11) | 315(17.1) | 1072(14.7) |
| 4 | 114(11.2) | 425(13.5) | 157(12) | 270(14.7) | 966(13.2) |
| 5+ | 215(21.2) | 1251(39.7) | 583(44.7) | 740(40.3) | 2789(38.1) |
| **Total** | 1,015 | 3,155 | 1,305 | 1,837 | 7,312 |

^1^ IQR=interquartile range

### Additional file 4.10: Factors associated with non-registration of a child surviving the neonatal period

|  | Births not registered | Birth registered | Unadjusted OR for non-registration (95%CI) | Adjusted OR for non-registration (95%CI)* |
| --- | --- | --- | --- | --- |
|  | N(%) | N(%) |  |  |
| **HDSS site** |  |  |  |  |
| Bandim | 1015 (77.1) | 302 (22.9) | 1 (ref) | 1 (ref) |
| Dabat | 3155 (93.9) | 206 (6.1) | 4.55 (0.38 – 5.50) | 4.11 (3.37 – 5.01) |
| IgangaMayuge | 1305 (68.1) | 612 (31.9) | 0.63 (0.54 – 0.74) | 0.57 (0.48 – 0.68) |
| Kintampo | 1837 (46.5) | 2,114 (53.5) | 0.26 (0.22 – 0.30) | 0.23 (0.20 – 0.27) |
| **Child sex** |  |  |  |  |
| Boy | 3619 (69.2) | 1,608 (30.8) | 1 (ref) | 1 (ref) |
| Girl | 3693 (69.4) | 1,626 (30.6) | 1.01 (0.93 -1.10) | 0.97 (0.88 – 1.07) |
| **Age at time of survey (months)** |  |  |  |  |
| <12 | 1878 (77.3) | 551 (22.7) | 1 (ref) | 1 (ref) |
| 12-23 | 1739 (67.2) | 848 (32.8) | 0.60 (0.53 – 0.68) | 0.66 (0.57 – 0.76) |
| 24-35 | 1466 (66.9) | 724 (33.1) | 0.59 (0.52 – 0.68) | 0.65 (0.56 -0.76) |
| 36-47 | 970 (64.6) | 531 (35.4) | 0.54 (0.46 – 0.62) | 0.61 (0.51 – 0.72) |
| 48 or more | 1251 (68.3) | 580 (31.7) | 0.63 (0.55 – 0.73) | 0.65 (0.55 – 0.76) |
| **Maternal Age** |  |  |  |  |
| 15-19 | 345 (77.4) | 101 (22.6) | 1 (ref) | 1 (ref) |
| 20-24 | 1,374 (73.0) | 507 (27.0) | 0.79 (0.62 – 1.01) | 0.82 (0.62 – 1.08) |
| 25-29 | 1,732 (69.5) | 760 (30.5) | 0.67 (0.53 – 0.85) | 0.63 (0.47 – 0.84) |
| 30-34 | 1,535 (67.1) | 753 (32.9) | 0.60 (0.47 – 0.76) | 0.54 (0.40 – 0.73) |
| 35+ | 2,323 (67.6) | 1,112 (32.4) | 0.62 (0.48 – 0.77) | 0.50 (0.36 – 0.69) |
| **Education** |  |  |  |  |
| No education | 3333 (77.8) | 952(22.1) | 1 (ref) | 1 (ref) |
| Primary Only | 2448 (63.9) | 1383(36.1) | 0.51 (0.46 – 0.56) | 0.78 (0.69 – 0.88) |
| Primary & secondary | 1192 (60.8) | 770(39.2) | 0.44 (0.39 – 0.50) | 0.70 (0.60 – 0.81) |
| Higher | 339 (72.4) | 129(27.6) | 0.75 (0.61 – 0.93) | 0.54 (0.41 – 0.70) |
| **Birthplace** |  |  |  |  |
| Health facility | 4291 (63.4) | 2478 (36.6) | 1 (ref) | 1 (ref) |
| Home birth | 3021(80.0) | 756 (20.0) | 2.31 (2.10 – 2.53) | 1.43 (1.27 – 1.60) |
| **Social economic status** |  |  |  |  |
| 1 (Poorest) | 2085 (77.9) | 591 (22.1) | 1 (ref) | 1 (ref) |
| 2 | 1549 (72.1) | 600 (27.9) | 0.73 (0.64 – 0.83) | 0.77 (0.67 – 0.90) |
| 3 | 1471 (72.2) | 567 (27.8) | 0.74 (0.64 – 0.84) | 0.73 (0.63 – 0.85) |
| 4 | 1234 (65.5) | 649 (34.5) | 0.54 (0.47 – 0.62) | 0.56 (0.48 – 0.66) |
| 5 (Richest) | 973 (54.1) | 827 (45.9) | 0.33 (0.29 – 0.38) | 0.39 (0.33 – 0.46) |
| **Parity** |  |  |  |  |
| 1 | 1255 (67.7) | 599 (32.3) | 1 (ref) | 1 (ref) |
| 2 | 1230 (66.7) | 613 (33.3) | 0.96 (0.83 – 1.10) | 1.08 (0.91 – 1.28) |
| 3 | 1072 (67.8) | 510 (32.2) | 1.00 (0.87 – 1.16) | 1.31 (1.08 – 1.59) |
| 4 | 966 (68.1) | 453 (31.9) | 1.02 (0.88 – 1.18) | 1.32 (1.07 – 1.63) |
| 5+ | 2789 (72.5) | 1059 (27.5) | 1.26 (1.11 – 1.42) | 1.44 (1.16 – 1.79) |

###

### Additional file 4.11: Reasons for non-registration for children surviving the neonatal period

796 responded yes to ‘other reasons for non-registration’. These free text responses were translated into English and coded into categories. 49 women provided the same reason as they had given in the response to the list of options for reasons for non-registration and were excluded.

747 women provided new reasons for non-registration, 86 of these responses were coded onto a category in the original response list, the remainder were coded and grouped into new categories, categories with ≥5 women reporting the same reason are shown below:

| **Reasons for non -registration in free text ‘other category’:** | Total |
| --- | --- |
| Not enough money to pay the cost of registering the baby | 4 |
| Father of baby is required but is unwilling/unable | 52 |
| Distance to registration center is far | 3 |
| Unable to produce full set of documents to register the baby | 27 |
| Administrative issues | 61 |
| Child is too young | 60 |
| Don't know anything about birth registration | 258 |
| Lack knowledge of how/ where to register the birth | 151 |
| Other reasons | 131 |
| *Child is sick or died* | *13* |
| *Mother is sick* | *10* |
| *Not seen as important* | *16* |
| *Not enough time to do this* | *36* |
| *Travelling* | *7* |
| *Other* | *49* |

These free text responses were combined with results from the list that was read to the mother:

(i) Birth registration is not a legal requirement, (ii) There is not enough money to pay the cost of registering (name), (iii) The distance to the registration center is far, (iv) The registration process is too complicated to understand, (v) I am unable to produce the full set of documents required to register (name), (vi) The father of (name) is required to be present but he is unable or unwilling to attend, (vii) The name of the child is required but it has not yet been given by the family.

See Figure 6 in main manuscript.

|  | Number selected from list | Number from free-text responses | Total | Percent of respondents |
| --- | --- | --- | --- | --- |
| Other reasons |  | 252 | 252 | 3.4 |
| Name of the baby is required but hasn't been given | 200 |  | 200 | 2.7 |
| Don't know anything about birth registration |  | 258 | 258 | 3.5 |
| Father of baby is required but is unwilling/unable | 409 | 52 | 461 | 6.3 |
| Birth registration not a legal requirement | 636 |  | 636 | 8.7 |
| Unable to produce full set of documents to register baby | 724 | 27 | 751 | 10.3 |
| Distance to registration center is far | 1177 | 3 | 1180 | 16.1 |
| Not enough money to pay the cost of registering the baby | 2076 | 4 | 2080 | 28.4 |
| Registration process is complicated to understand | 2486 | 151 | 2637 | 36.1 |
