# Supplementary material for: Birth, stillbirth and death registration data completeness, quality and utility in population-based surveys: EN-INDEPTH study
Source: Popul Health Metr. 2021 Feb 8;19(Suppl 1):14. doi: 10.1186/s12963-020-00231-2 (PMC7869445; doi:10.1186/s12963-020-00231-2)
Supplement: Supplementary file 5 — Additional file 5. Gap analysis for birth registration in EN-INDEPTH survey, by site. [file 12963_2020_231_MOESM5_ESM.docx]

## Additional File 5: Gap analysis for birth registration in EN-INDEPTH survey, by site

### Additional file 5.1A: Bandim


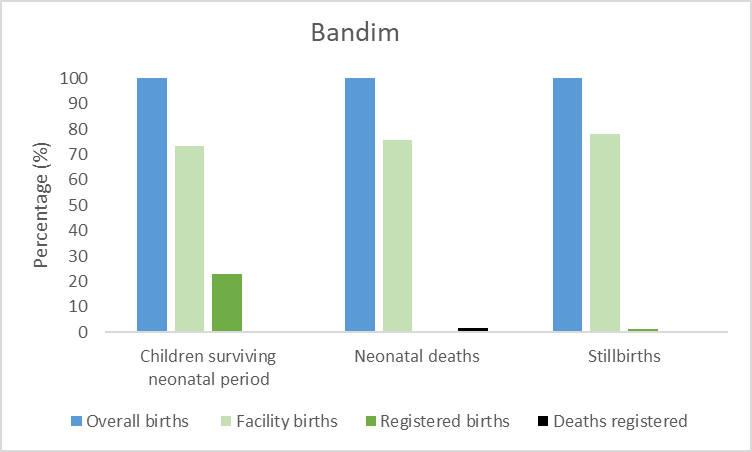


### Additional file 5.1B: Dabat


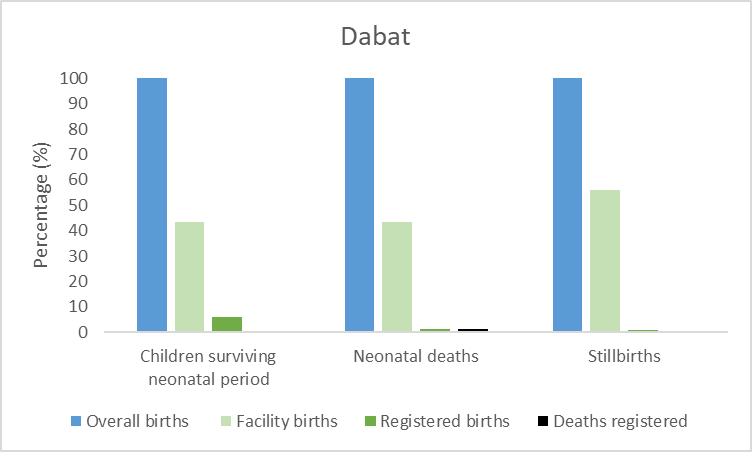


### Additional file 5.1C: IgangaMayuge


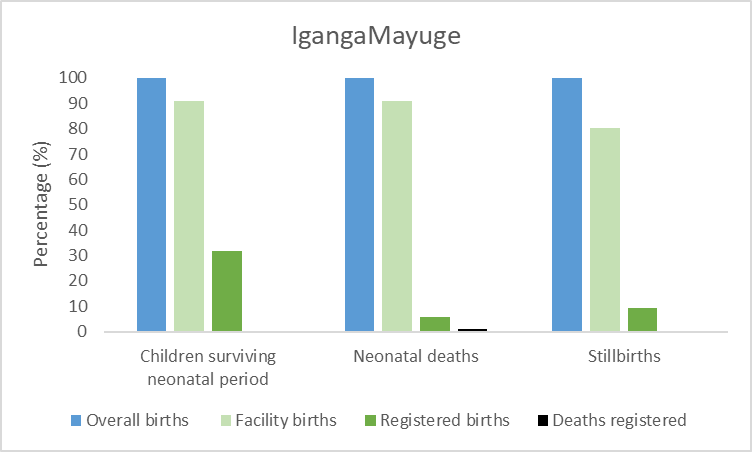


### Additional file 5.1D: Kintampo


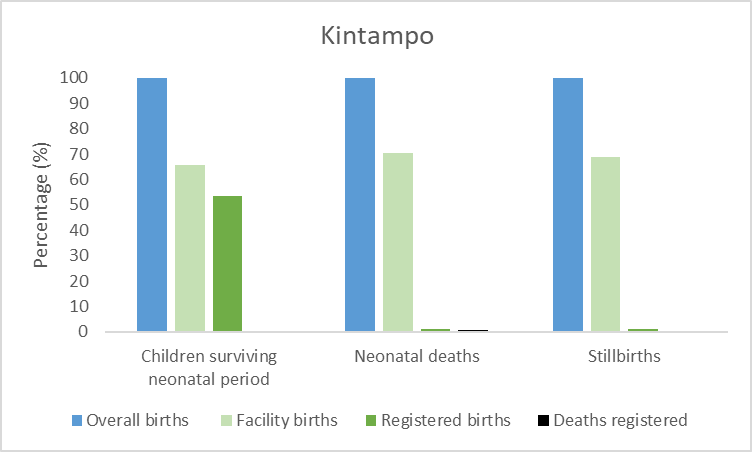


### Additional file 5.2: Overall percent of facility births registered

|  | % of facility births registered |
| --- | --- |
| Overall | 47.8 |
| Bandim | 31.2 |
| Dabat | 14.0 |
| IgangaMayuge | 35.1 |
| Kintampo | 81.3 |
